# Larval Defense against Attack from Parasitoid Wasps Requires Nociceptive Neurons 

**DOI:** 10.1371/journal.pone.0078704

**Published:** 2013-10-25

**Authors:** Jessica L. Robertson, Asako Tsubouchi, W. Daniel Tracey

**Affiliations:** 1 Department of Cell Biology, Duke University, Durham, North Carolina, United States of America; 2 Department of Anesthesiology, Duke University Medical Center, Durham, North Carolina, United States of America; 3 Department of Neurobiology, Duke University, Durham, North Carolina, United States of America; Alexander Fleming Biomedical Sciences Research Center, Greece

## Abstract

Parasitoid wasps are a fierce predator of *Drosophila* larvae. Female *Leptopilina boulardi* (LB) wasps use a sharp ovipositor to inject eggs into the bodies of *Drosophila melanogaster* larvae. The wasp then eats the *Drosophila* larva alive from the inside, and an adult wasp ecloses from the *Drosophila* pupal case instead of a fly. However, the *Drosophila* larvae are not defenseless as they may resist the attack of the wasps through somatosensory-triggered behavioral responses. Here we describe the full range of behaviors performed by the larval prey in immediate response to attacks by the wasps. Our results suggest that *Drosophila* larvae primarily sense the wasps using their mechanosensory systems. The range of behavioral responses included both “gentle touch” like responses as well as nociceptive responses. We found that the precise larval response depended on both the somatotopic location of the attack, and whether or not the larval cuticle was successfully penetrated during the course of the attack. Interestingly, nociceptive responses are more likely to be triggered by attacks in which the cuticle had been successfully penetrated by the wasp. Finally, we found that the class IV neurons, which are necessary for mechanical nociception, were also necessary for a nociceptive response to wasp attacks. Thus, the class IV neurons allow for a nociceptive behavioral response to a naturally occurring predator of *Drosophila*.

## Introduction

The evolutionary arms race produces an ever-changing range of predatory behaviors and defensive prey responses. Indeed, predator-prey interactions are an important evolutionary force. While predator and prey behaviors have been characterized on a macro-scale, essentially nothing is known about the genetic basis of these behaviors, many of which are innate and encoded by the genomes of the predator and the prey. Parasitoid wasps are a common predator of *Drosophila* larvae, and can infect up to 70% of larvae in the wild [1]. These wasps inject eggs into the body of *Drosophila* larvae [2], which after hatching, proceed to eat the *Drosophila* larva from the inside [3]. The predatory adaptations that parasitoid wasps use to find and infect *Drosophila* larvae, including search strategies, ovipositor properties, and immunosuppressive mechanisms, are well described [4-16]. However, little is known about the behavioral responses of the *Drosophila* larvae to parasitoid attack [15,17,18]. Given the powerful genetic tools available to *Drosophila* researchers, this system represents an opportunity for the study of predator-prey interactions in unprecedented detail. 

Previous studies have shown that *Drosophila* larvae show nocifensive escape locomotion, a corkscrew-like rolling around the anterior/posterior axis, in response to noxious thermal or mechanical stimulation [18-22]. A single class of neuron, the multi-dendritic class IV neuron, is both necessary and sufficient for triggering nocifensive escape locomotion [18], and thus have been functionally defined as nociceptors. Within each larval hemi-segment, there are three different class IV neurons, localized to the dorsal, lateral, and ventral region (named ddaC, v’ada, and vdaB, respectively) [23]. The class IV neurons possess elaborately branched dendritic arbors [23-30] which tile the larval body wall [23,25]. Similar to nociceptors in vertebrates, the class IV neuron dendrites are “naked” as they are not known to be associated with other receptor cells. In *Drosophila*, these dendrites are attached to the epidermal basal lamina and are partially ensheathed by overlying epidermal cells [31-33]. 

In a previous study it was shown that like optogenetic activation of class IV neurons, attack of parasitoid wasps also triggers nocifensive escape behavior [18]. This led to the proposal that larval nocifensive escape locomotion may have evolved as an adaptation to protect against parasitoid wasps. Here, we explore this idea further. First, we describe the full range of behaviors shown by larvae in response to wasp attacks. We find that the location of the attack, as well as the penetration of the larval cuticle, determine which type of response the larvae exhibit. Next, we explore the role of the class IV neurons in mediating the behavioral response to the wasp attack, and find that the class IV neurons are necessary for nocifensive escape locomotion following the wasp attack. Finally, we measure the mortality of larvae that produce nocifensive responses and our results suggest that nocifensive behavior leads to escape from 50% of wasp attacks. Our study establishes a new paradigm for observing and genetically manipulating predator - prey interactions.

## Results and Discussion

### *Drosophila* larvae show multiple stereotyped behaviors in response to attacks by *Leptopilina boulardi*

To better understand this system we observed the defensive behaviors performed by *Drosophila* larvae in response to attacks by parasitoid wasps. The larval prey (40-50) were first placed in small agar containing petri dishes that were lightly coated with conditioned yeast paste (see Materials and Methods). The latter provided important chemosensory cues to stimulate egg-laying by the wasps [6]. Next, 2-3 mated *Leptopilina boulardi* wasps (a well characterized, specialist parasitoid of *Drosophila melanogaster* [13,34]) were placed in the petri dish with the larvae, and all wasps were observed until one of the wasps began attacking. We then followed the actively attacking wasp for a period of 10 minutes while videorecording the interactions between the wasp and the larvae. Interestingly, prior to contact with the wasps, larvae appeared to be unable to sense their presence. Larvae did not alter their course of locomotion despite the nearby presence of the wasps and they would often crawl right up to them, even bumping directly into their legs. However, once physical contact with the wasp was made, larvae did show responses that resembled previously described mechanosensory behaviors [18,22,35-37]. 

**Figure 1:**
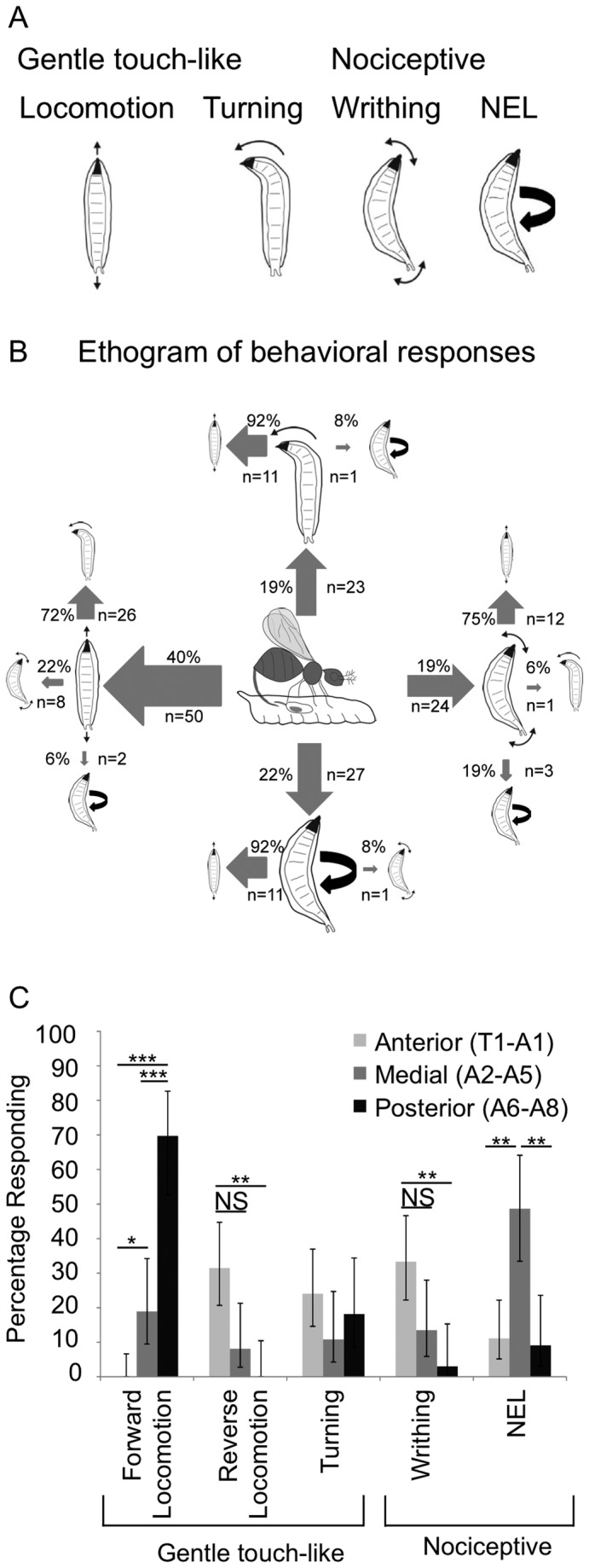
Behavioral responses of *Drosophila melanogaster* larvae to attack by LB. (A) Classification of behavioral responses to attacks by parasitoid wasps. The cartoons depict peristaltic locomotion, turning, writhing and nocifensive escape locomotion ((NEL) also see supplemental movies). (B) Ethogram of behaviors shown by third instar larvae (based on observations of 124 attacks). The size of the arrow is weighted according to the observed frequency of the behavior. Primary behaviors are indicated by large cartoons, and secondary behaviors are indicated by the smaller cartoons. Tertiary behaviors are not shown. (C) Attack position along the larval body wall influences behavioral response. Fisher’s Exact Test with Holm-Bonferroni correction. Data are presented as percentages ± 95% confidence intervals. P< .05 =*, P<.01=**, P< .001 =***. N=54(Anterior), N=37(Medial), N=33(Posterior).

Several distinct types of responses to the wasp oviposition attempts were seen. Larvae displayed peristaltic locomotion in either the forward or the reverse direction (Movie S1, Figure 1A) and/or rapid turning responses (Movie S2, Figure 1A). The persistaltic locomotion responses and the turning responses are also seen in so-called “gentle touch assays” in which larvae are touched with an eyelash by an investigator [35,36]. In addition, as described in a previous study, we also observed nociceptive-related behaviors, including writhing (turning of either the anterior or posterior back and forth) (Movie S3, Figure 1A) and nocifensive escape locomotion (Movie S4, Figure 1A) [18-22]. Thus, stimulation of the larvae by the wasp ovipositor causes a variety of mechanosensory behaviors that include both gentle touch-like and nociceptive behaviors. 

In over half of attacks, larvae responded by producing a sequence of the different behaviors while in the remainder of attacks only a single response was observed. In Figure 1B an ethogram describing the frequency and order with which each behavior occurred is shown with the behaviors categorized as either primary or secondary responses (Figure 1B). The most commonly observed primary response was peristaltic locomotion, which occurred in 40% of attacks (Figure 1B). Turning, writhing, and nocifensive escape locomotion each occurred at approximately equal frequency, and were the primary response in about 20% of the attacks (Figure 1B). 

As with the primary response, peristaltic locomotion was the most frequent secondary response, and was observed after turning, writhing, or nocifensive escape locomotion (Figure 1B). Peristaltic locomotion was equally likely to occur as a primary (40%) or secondary behavior (45%). Writhing was also equally likely to occur as a primary (19%) or secondary (12%) behavior. Turning behavior tended to occur more as a secondary response (36%) than a primary response (19%, p<.05, Fishers Exact Test with Holm-Bonferroni correction). Lastly, nocifensive escape locomotion occurred most frequently as a primary response (22%), and more rarely as a secondary response (8%, p<.05, Fishers Exact Test with Holm-Bonferroni correction). 

 We noticed that some of the variation in the larval behavioral responses appeared related to the location of the wasp attack along the larval body. As shown in Figure 1C, this observation was supported by further analysis. Wasps attacked the anterior segments (T1-A1) 54 times, the medial segments (A2-A5) 37 times and the posterior segments (A6-A8) 33 times. Forward locomotion occurred most frequently when the wasp attacked in the posterior region, and reverse locomotion or writhing happened most frequently when the wasp attacked in the anterior region (Figure 1C). Interestingly, nocifensive escape locomotion was much more likely to occur when the attack was in the medial segments (Figure 1C). 

Note that the frequency of forward locomotion as a response was likely to be overestimated. This is because many of the larvae (30%) that were scored as showing forward locomotion were already engaging in forward locomotion at the moment when they were first attacked. Thus, in these attacks we were unable to discern whether the forward locomotion that we observed was an actual response to the attack, or if the larvae had simply continued their previous behavioral pattern. Forward locomotion may be an important means of escape for larvae that are burrowed into a fruit in the wild. Consistent with this there were several instances where we observed a burrowed larva that was attacked in the posterior and the larval forward locomotion response was seen to pull the wasp down into the burrow. Because the wasp ovipositor has a limited length, it is possible that this response would cause the wasp to disengage its attack prior to egg-laying.

### Attacks with nocifensive Responses Show Greater Penetration of the Larval Cuticle

**Figure 2:**
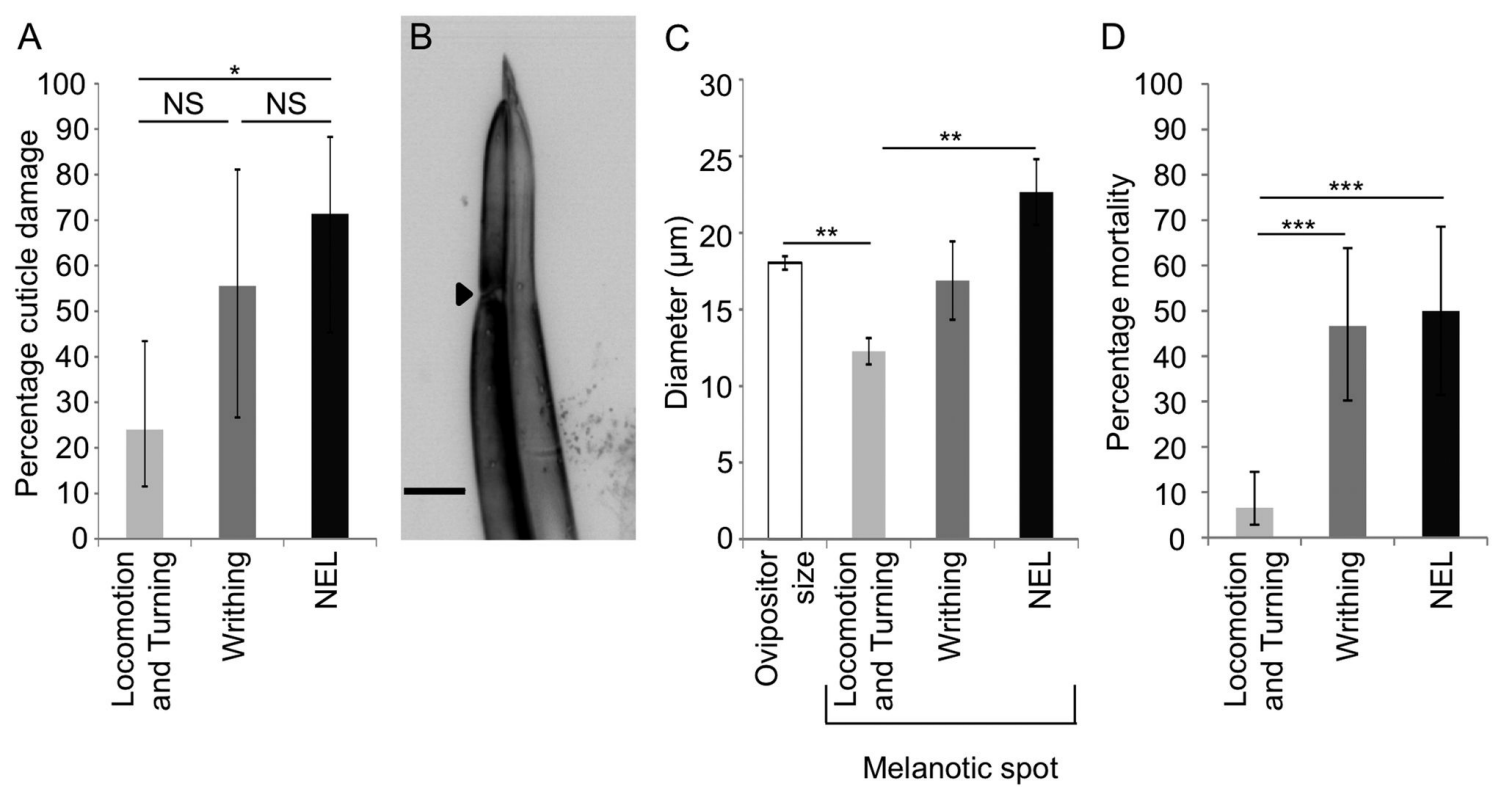
Cuticle penetration and mortality is more frequent in attacks with nociceptive behaviors. (A) Larvae that showed nocifensive escape locomotion showed frequent penetration of the cuticle (N=14, 71% (+17/-26)). Gentle touch-like behaviors (turning and locomotion) rarely showed penetration to the cuticle (N=25, 24% (+19/-13)). Writhing behaviors were associated with an intermediate level of penetration (N=9, 56%, (+26/-29)). Fisher’s Exact Test with Holm-Bonferroni correction. Data are presented as percentages ±95% confidence intervals. (B) Representative photomicrograph of a wasp ovipositor (scale bar=20 µm). The arrowhead indicates the location of the ovipositor clip. (C) The melanotic spot was similar to the diameter of the ovipositor clip (N=12, 18µm, ± .4) when attacks triggered either writhing (N=5, 17µm, ±2.6) or nocifensive escape locomotion (N=9, 23µm, ±2.2). The size of the melanotic spot was smaller than the diameter of the ovipositor clip when larvae that showed non-nociceptive behaviors (N=6, 12µm, ±.9). T-test with Holm-Bonferroni correction. Error bars denote standard error of the mean. (D) Mortality was high in larvae that displayed writhing (N=30, 47%, (+17/-16)) or nocifensive escape locomotion (N=24, 50%, (+19/-19)) relative to larvae that displayed locomotion and turning (N=76, 7%, (+8/-4)) . Fisher’s Exact Test with Holm-Bonferroni correction. Data are presented as percentages ±95% confidence intervals. P<.05=*, P<.01=**, P< .001 =***.

Our above observation of wasp attacks and larval behavior did not provide information on whether the wasps successfully penetrated the larval cuticle in a particular attack. Thus, the observation of larval behaviors does not provide any specific information on the degree to which a particular behavior might provide a selective advantage to the larvae. Nevertheless, because successful epidermal penetration by the wasps triggers a melanization cascade in the larvae, and this leaves a “melanotic spot” that is visible under a microscope [38], we were able to directly test whether specific attacks resulted in penetration. To achieve this, we observed larvae being attacked by wasps, recorded the larval behavioral response, and later searched for the presence or absence of a melanotic spot while simultaneously observing the nociceptor dendrites under a confocal microscope. These analyses revealed that successful cuticle penetration occurred in only 44% of all wasp attacks. Cuticle penetration was relatively rare in the attacks that resulted in non-nociceptive behaviors, occurring only 24% of the time (Figure 2A). Larvae that showed writhing behavior had an intermediate frequency of cuticle penetration (56%) (Figure 2A). Interestingly, larvae that performed nocifensive escape locomotion following attacks showed the highest frequency of cuticle penetration (71%) (Figure 2A). The cuticle penetration of larvae that showed nocifensive responses was primarily confined to the medial body segments (78% of the time) further confirming the importance of somatotopy in determining the behavioral responses of larvae. 

The wasp ovipositor possesses a specialized structure that resembles the barb of a fish hook. It is believed that this structure, termed the clip, prevents removal of the ovipositor from the struggling larvae during the attack (Figure 2B) [11,17]. Interestingly, when the attack resulted in nociceptive related responses, the diameter of the melanotic spot was similar to the diameter of the ovipositor clip which is consistent with the idea that the ovipositor penetrated to the depth of the clip in these attacks (Figure 2C). In contrast, in attacks that elicited gentle touch-like behaviors the diameter of the melanotic spot was significantly smaller than the wasp ovipositor clip which suggests that these attacks resulted in only partial penetration (Figure 2C). These data are consistent with the previously proposed function for the ovipositor clip [11,17] and they further suggest that the most vigorous escape responses in the larvae actually occur primarily in those attacks that involve successful and deep penetration of the larval epidermis. Thus, the ovipositor clip may have evolved as an adaptation against the nocifensive responses. 

### Larval mortality following different behavioral responses

The data above indicate that wasps most successfully penetrated the cuticle when attacking medially and that the penetrating attacks were the most likely to elicit nocifensive behavior. Is the nocifensive escape locomotion also an effective means of escape following cuticle penetration? If this were so, we would expect that larval mortality in attacks that elicited nocifensive escape locomotion would be lower than the penetration rate for these attacks (71%). Consistent with this hypothesis we found that the mortality of larvae that showed nocifensive escape locomotion behavior during wasp attacks was 50% (Figure 2D, Materials and Methods). Because *Drosophila melanogaster* is not capable of mounting a successful immune response to this strain of *Leptopilina boulardi*, the difference observed between the frequency of cuticle penetration and mortality likely indicates that larvae are able to escape from wasps prior to oviposition when performing nocifensive escape locomotion.

While it is tempting to speculate on the efficacy of each of the different behavioral responses as a method of escape from the wasp, these comparisons are difficult. For example, low levels of cuticle penetration and low mortality were found with attacks that elicited gentle touch-like behaviors. One interpretation of these findings is that these behaviors are highly effective methods of escape and they thus prevent the wasp from penetrating the cuticle. Alternatively, the forces applied by the wasps in these particular attacks may merely be insufficient for penetrating the cuticle and/or triggering nocifensive responses. 

### Sparse activation of the class IV neurons causes nocifensive escape locomotion

**Figure 3:**
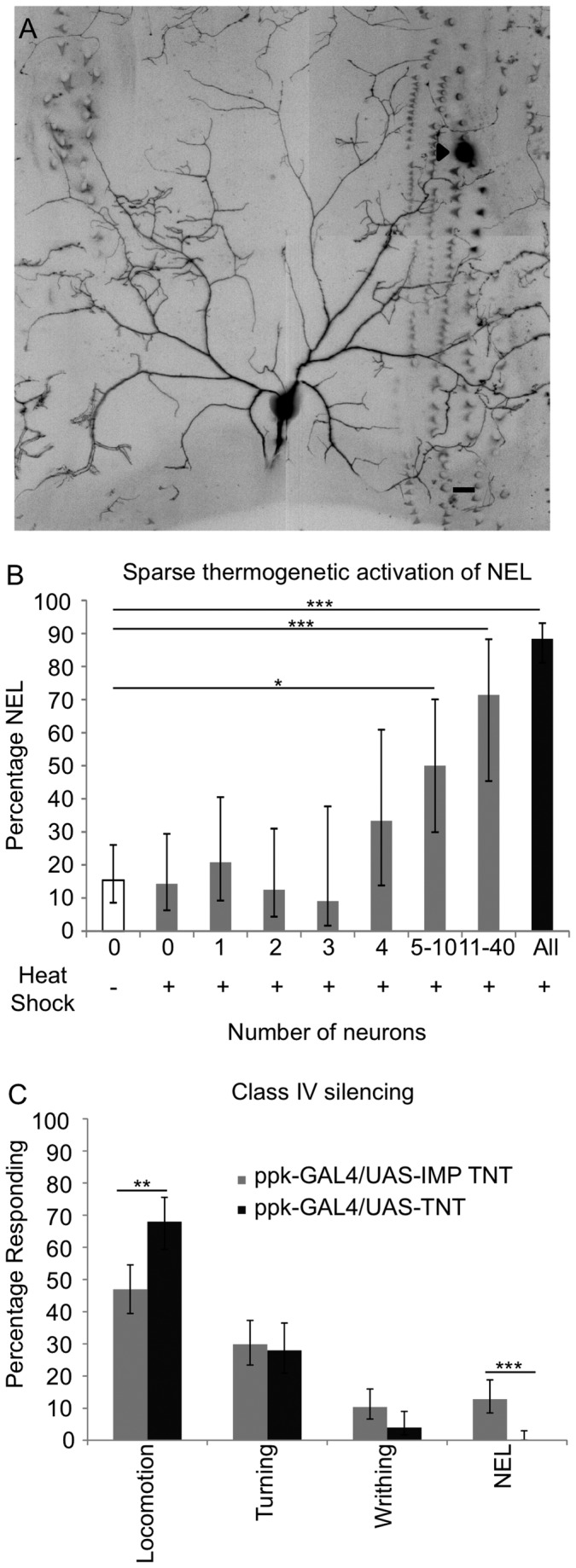
Role of the class IV neurons and nocifensive escape locomotion in response to wasp attack. (A) Confocal micrograph of the dendritic field of the dorsal (ddaC) class IV neuron taken from a larva (ppk-GAL4 UAS-mCD8::GFP/+) that displayed nocifensive escape locomotion following wasp attack. Scale bar is 20 μm. The location of ovipositor penetration is denoted by an arrowhead. See also Figure S1. (B) Expression of dTRPA1-A in 5-10 class IV neurons is sufficient to cause nocifensive escape locomotion. (0 neurons in the no heat shock control (N=65, 15%, (+11/-7)), 0 neurons following heat shock (N=35, 14% (+15/-8)), 1 neuron (N=24, 21%, (+20/-12)), 2 neurons (N=24, 13%, (+19/-8)), 3 neurons (N=11, 9%, (+29/-7)), 4 neurons (N=12, 33%, (+28/-20)), 5-10 neurons (N=20, 50% (+20/-20)), 11-40 neurons (N=14, 71%, (+17/-26)), positive controls (N=112, 88%, (+5/-7)). The genotype used was *w;hs-flp/pickpocket1.9-GAL4, UAS-dTRPA1-A;tub>GAL80>*/*UAS-mCD8::GFP*. For positive controls the genotype was *pickpocket1.9-GAL4, UAS-dTRPA1-A/+; UAS-mCD8::GFP/+*. (C) Larvae with class IV neurons silenced by *UAS-TNT-E* (N=125) show no nocifensive escape locomotion (0%, (+3/-0)) and increased locomotion (68%, (+8/-9)) compared to larvae expressing impotent TNT in the class IV neurons (N=164, nocifensive escape locomotion 13%, (+6/-4), locomotion 47%, (+8/-7)). The genotypes used were *w;ppk-GAL4/UAS-TNT*, *w;ppk-GAL4/UAS-IMP* *TNT*. Fisher’s Exact Test with Holm-Bonferroni correction. Data are presented as percentages ±95% confidence intervals. P<.05=*, P<.01=**, P< .001 =***.

Behavioral assays that trigger nocifensive escape locomotion are used for studies on the cellular and molecular mechanisms of nociception in *Drosophila* larvae [18,20-22,39]. We targeted the nocifensive escape locomotion response for further investigation so we could further understand this behavior in the context of an ecologically relevant stimulus. The class IV neurons are the primary nociceptors of *Drosophila* larvae and have an elaborate dendritic field which tiles the larval body wall [23-28,31]. Within each larval hemi-segment, there are three identifiable class IV neurons (ddaC, v’ada, and vdaB), which are localized to the dorsal, lateral, and ventral region, respectively [23]. Previous experiments have shown that either optogenetic or thermogenetic activation of class IV neurons is sufficient to trigger nocifensive escape locomotion [18,40]. The neuronal activators used in these approaches have been expressed in all of the class IV neurons and it thus remains unknown whether activation of a limited number of cells is sufficient for triggering the behavioral response. The wasp system presents a useful paradigm to investigate the circuitry of the nocifensive escape locomotion response due to the small size of the wasp ovipositor (Figure 2B and 2C) and to the ability to visualize the point of insertion after the attack. Interestingly, in our examination of melanotic spots described above, we found that the melanotic spot could be found within a single dendritic field of either the dorsal, the lateral, or the ventral class IV neuron (Figure 3A, Figure S1A, B). 

 Although the injury revealed by the melanotic spots was confined to the dendritic field of a single class IV neuron, it remained possible that the force generated by the insertion of the wasp ovipositor could be sensed by a more distributed population of neurons. Thus, we wished to determine the minimum complement of class IV neurons whose activation would be sufficient to cause nocifensive escape locomotion. To achieve this, we used an approach which allowed us to thermogenetically activate a small random subset of the class IV neurons. We first exposed animals of the genotype *w*;*hs-flp* /*pickpocket1*.*9-GAL4, UAS-dTRPA1-A;tub>GAL80>*/ *UAS-mCD8::GFP* to a 30 minute heat shock [41]. This caused expression of GAL4 in a random subset of the class IV neurons, which in turn caused expression of the warmth activated dTRPA1-A [40,42-45] channel and a fluorescent plasma membrane marker (mCD8::GFP). We then placed larvae in a 50 µL water droplet heated to 32°C, and observed the larval behavior for 10 seconds. Following the behavioral observations, larvae were mounted for microscopy and the class IV neurons expressing mCD8::GFP (as a proxy for GAL4 driven dTRPA1-A expression) were then identified. 

These experiments indicated that 5-10 GAL4 expressing neurons were needed to trigger nocifensive escape locomotion above the background level seen in control animals (without detectable expression of mCD8::GFP (Figure 3B)). These results indicate that although the wasp ovipositor is capable of triggering nocifensive escape locomotion when penetrating a single dendritic field, expression of dTRPA1-A in a single class IV neuron was not sufficient to cause nocifensive escape locomotion in response to warm temperatures under these experimental conditions. 

Although these results must be interpreted in the context of technical limitations of this approach (for example it is possible that the expression levels of dTRPA1-A in this experiment are too low to reliably induce activation of every neuron that expresses GFP) a more intriguing interpretation is that the penetration by the wasp might activate a small population of class IV neurons even though the ovipositor damage is confined to the field of a single neuron. This could occur through viscoelastic coupling of the forces across several segments of the larvae, or alternatively, damage to epidermal cells may allow the damage signal to spread to many neurons at once. The latter possibility is consistent with the results of recent studies which indicate that the nociceptor dendrites are ensheathed by epidermal cells [32,33] and with the previously described electrical coupling of epidermal cells that occurs through gap junctions [46].

### The class IV neurons are necessary for nocifensive escape locomotion in response to wasp attacks

 The class IV neurons are necessary for nocifensive escape locomotion in response to artificially applied noxious thermal or mechanical stimuli [18]. However, the wasp attack represents a qualitatively distinct stimulus, with the potential for breaking through the cuticle, epidermal cells, and possibly the dendrites of the nociceptors. Thus, we were interested in determining whether the class IV neurons were indeed required for nocifensive responses to wasp attacks. To test this possibility, we silenced the class IV neurons through expression of tetanus toxin light chain (TNT) [47] and investigated the effects on the larval response to wasp attacks. As expected if the nociceptive neurons were specifically involved in sensing the harshest of attacks, larvae with silenced class IV neurons showed an abolishment of nocifensive escape locomotion (Figure 3C). This indicates that the class IV neurons are responsible for mediating the nocifensive escape locomotion response to wasp attacks. We also noted that there was an apparent increase in the peristaltic locomotion response in these animals. This may indicate that “gentle touch” pathways are still engaged during attacks that would normally have triggered nocifensive responses in an intact animal. Interestingly, larvae mutant for the *pickpocket* gene, which is expressed highly in the class IV neurons, have been reported to show a higher base-line level of locomotor activity [48]. Thus, the increased locomotion response in the wasp response could reflect a similar effect.

 In conclusion, *Drosophila* larvae show a variety of mechanosensory behaviors in response to attacks by parasitoid wasps. These responses are strongly influenced by the somatotopic location of the attack. Attacks with nocifensive responses showed the highest frequency of cuticle penetration and penetration of a single nociceptor field was sufficient to trigger nocifensive responses. Nevertheless, expression of dTRPA1 in a population of 5-10 nociceptive neurons was needed to trigger nocifensive escape locomotion. Interestingly silencing of class IV md-da neurons eliminated nocifensive escape locomotion responses to wasp attacks, which demonstrates that the neuronal pathways identified with artificial stimuli also play a role in escape from a natural deadly stimulus. Our thorough characterization of the interactions between *Drosophila* larvae and parasitoid wasps open up the field of predator-prey interactions and will allow for a detailed analysis of the genomic encoding of prey escape behavior. 

## Materials and Methods

### Fly and Wasp Strains and Husbandry

The following fly strains were used: Canton S, *w*;*pickpocket1*.*9-GAL4*, *UAS-mCD8::GFP, w*;*hs-flp*;*tub>GAL80>*, *w*;*pickpocket1*.*9-GAL4 UAS-dTRPA1-A; UAS-mCD8::GFP/K87(T(2:3 SM6a:TM6b*)*Cy Tb Hu, w*;*pickpocket1*.*9-GAL4, w*;*UAS-TNT (E*)*, w*;*UAS-IMP TNT(V*). Flies were maintained on standard cornmeal molasses medium at room temperature. *Leptopilina boulardi-17* were kept in fly vials at room temperature and fed by placing several drops of a 50% honey solution on the vial plug. Wasp strains were propagated by first allowing Canton S flies to lay eggs for 1-3 days on standard molasses cornmeal medium. After removing the flies, male and female wasps were added to the vial and allowed to parasitize the fly larvae for 1-3 days. The infected vials were kept at room temperature until the wasps emerged, 3-4 weeks later. Once the wasps began to eclose, in order to minimize potential contact with larvae, vials were emptied of wasps once per day. Mated naïve wasps aged 3-12 days were used for all experiments. 

### Wild Type Behavioral Assays

Approximately 40 female and 20 male Canton S flies were allowed to lay eggs for 3-3.5 hours on agar apple juice plates with a small amount of yeast paste at 25°C. Behavioral assays were conducted 72.5 -76 hours post egg lay. 50 larvae were placed on a 30 mm petri dish containing 1% agar and a small amount of conditioned yeast paste from the agar apple juice plate. This yeast contained the larval kairomones which are necessary to activate the wasp oviposition behavior [5]. 2-3 wasps were placed in the petri dish with the larvae and allowed to acclimate. Video recording through the stereomicroscope began when a wasp began ovipositing. 

### Class IV Silencing Assays

For the class IV silencing experiment, 40-50 virgin females (genotype *w*;*ppk-GAL4*) were crossed with 20 males (genotype *w*;*UAS-TNT*, or *w*;*UAS-IMP TNT*). Flies were allowed to lay eggs for 3-3.5 hours on agar apple juice plates with a small amount of yeast paste at 25°C. Behavioral assays were then performed as described above, with the exception of using a 3% agar plate, and 40 larvae. 

### Behavioral Analysis

 After an acclimation period, wasp behavior was monitored for 10 minutes from the first clear attack. Larval behavior was monitored during the attack. Any larva for which the behavior during the attack could not be clearly visualized was excluded from the study, including any larva that was burrowed during the attack. Any trial with less than 5 wasp attacks during the 10 minute period was excluded from the study. Lastly, if the larva was completely motionless the attack was excluded. In some instances (20% of attacks), multiple attacks occurred on the same larva, and any time the wasp changed its position along the larval body wall was counted as a new attack. Nocifensive escape locomotion was scored if the larvae performed a complete 360° around the anterior/posterior axis. Partial rotations and/or the repeated back and forth movement of the anterior or posterior were scored as writhing. Turning was scored if either the anterior or posterior moved to one side. Peristaltic locomotion was scored when a peristaltic wave of locomotion propelled the larvae in either the forward or reverse direction. Behavioral assays were carried out on several different days, and the results were pooled, after which we performed the Fisher’s Exact Test. For Figure 1C and 3C only the first response to each attack was utilized for analysis. In the class IV silencing experiment, behavioral analysis was performed with coded samples that blinded the experimenter to the genotype of the larvae. For Figure 1B, the percentage of primary behaviors was calculated by dividing the number of behavioral occurrences within a category by the total number of primary behaviors. For the secondary behaviors, the percentage was calculated by dividing the total number of times the behavior was seen as a secondary behavior by the total number of secondary behaviors.

### Cuticle Penetration Assay

 Approximately 40 virgin *w*;*pickpocket1*.*9-GAL4 UAS-mCD8::GFP* females were crossed with 20 CS males and allowed to lay eggs for 3-3.5 hours on agar apple juice plates with a small amount of yeast paste at 25°C. Approximately 70 hours after egg lay, larvae were placed on a 1% agar plate with a small amount of yeast paste and 2-3 wasps. When one of the wasps began injecting, the other wasps were removed to ensure that all attacks could be observed. After observing the attack the larvae were removed from the plate to an eppendorf tube, which had an air hole poked through the top, and yeast paste for the larva to eat. The location of the injection and the behavioral response were noted on the tube. The next day, larvae were anesthetized with ether and imaged using a 40X oil immersion lens on a Zeiss LSM 5 live confocal microscope. The diameter of the melanotic spot was measured at the widest point using the distance tool of the Zeiss confocal software package.

### Larval mortality assay

The same behavioral protocol was followed as outlined in the cuticle damage assay. Larvae were removed from the arena following the attack and pooled (according to the behavior shown (motion, turning, writhing or nocifensive escape locomotion)) into fly food vials containing yeast paste. The flies that eclosed from each group were counted and mortality was determined by subtracting the number of flies that eclosed from the total number of pupal casings in the vial. 

### Single Cell Activation Assay

50 virgin females of the genotype *w*;*hs-flp*;*tub>GAL80*> were crossed to 10-20 males of the genotype *w*;*pickpocket1*.*9-GAL4 UAS-dTRPA1-A; UAS-mCD8::GFP/K87 Tb* for the negative controls and experimental group. For the positive controls, 50 virgin CS flies were crossed to 10-20 males of the genotype *w*;*pickpocket1*.*9-GAL4 UAS-dTRPA1-A; UAS-mCD8::GFP/K87 Tb*. The flies were allowed to lay eggs on apple juice plates for 2-2.5 hours at 25°C. Three hours after egg lay, embryos were heat shocked in a hot water bath at either 35 or 37°C for 30 minutes. Two temperatures were used in order to increase the variability in the number of neurons expressing GAL4. Negative controls were not heat shocked. Four days later, larvae were placed one at a time in a 50 µl water droplet on a hot plate set to 32.5°C, which heated the water droplet to a temperature of 31.7°C as measured by a fine thermocouple probe (IT-23, Physitemp). The larval behavior was observed for 10 seconds. After the behavioral assay, larvae were placed in PBS and a small cut was made to segment A8 so that the digestive tract could be removed with forceps. Larvae were mounted between coverslips in PBS and the class IV neurons expressing mCD8::GFP were counted and identified on a Zeiss LSM 5 Live microscope using a 20X objective. For the no heat shock controls, larvae were also visualized after the behavioral experiment to rule out leaky expression of the heat shock FLPase in the parental gametes. Larvae in which all of the class IV neurons were expressing mCD8::GFP (which occurs due to leaky heat shock FLPase expression) were excluded from the analysis. 

## Supporting Information

Figure S1 **Damage to the lateral and ventral class IV neurons can induce nocifensive escape locomotion.** Confocal micrograph of the dendritic field of the (A) ventral (vdaB) and (B) lateral (v’ada) class IV neurons taken from larvae (ppk-GAL4,UAS-mCD8::GFP/+).that displayed nocifensive escape locomotion following wasp attack. Scale bars are 20 μm. The location of ovipositor penetration is denoted by the arrowhead.
(TIF)

Movie S1 **Representative movie of peristaltic locomotion.** In the first segment of the movie, the larva is attacked in the posterior, resulting in forward locomotion. In the second segment, the larva is attacked in the anterior, resulting in reverse locomotion.
(MOV)

Movie S2 **Representative movie of turning.** In the first segment of the movie, the wasp attacks the larvae in the anterior, resulting in turning. In the second segment, the larva is attacked in the posterior, resulting in turning.
(MOV)

Movie S3 **Representative movie of writhing.** The wasp attacks the larva in the anterior, resulting in writhing. After the wasp disengages, the larva shows NEL.
(MOV)

Movie S4 **Representative movie of nocifensive escape locomotion.** The wasp attacks the larva in the medially, resulting in NEL.
(MOV)
