# Supplementary figures and images for: Larval Defense against Attack from Parasitoid Wasps Requires Nociceptive Neurons
Source: PLoS One. 2013 Oct 25;8(10):e78704. doi: 10.1371/journal.pone.0078704 (PMC3808285; doi:10.1371/journal.pone.0078704)

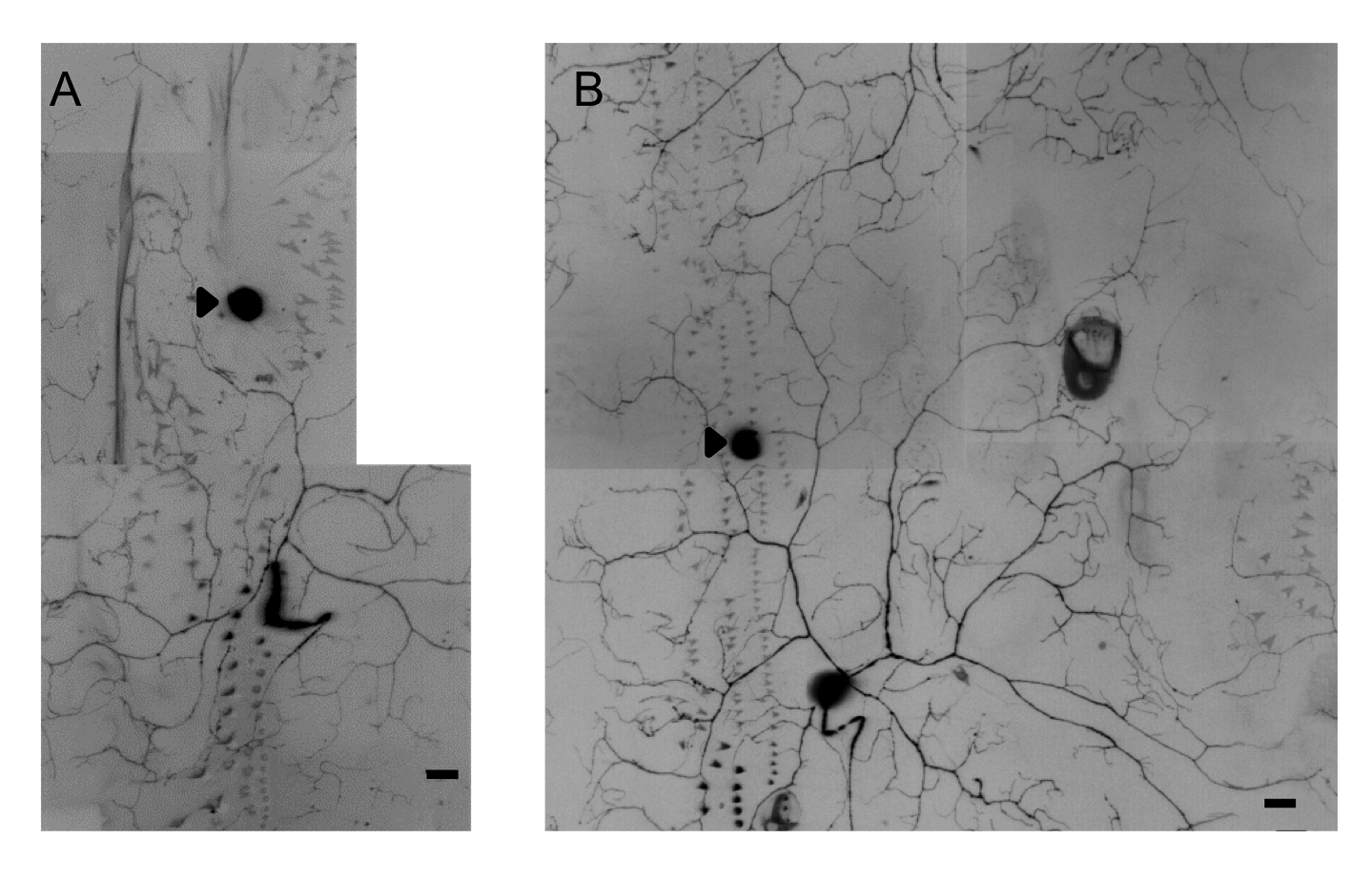

Supplement: Figure S1 — Damage to the lateral and ventral class IV neurons can induce nocifensive escape locomotion. Confocal micrograph of the dendritic field of the (A) ventral (vdaB) and (B) lateral (v’ada) class IV neurons taken from larvae (ppk-GAL4,UAS-mCD8::GFP/+).that displayed nocifensive escape locomotion following wasp attack. Scale bars are 20 μm. The location of ovipositor penetration is denoted by the arrowhead. (TIF) [file pone.0078704.s001.tif]
